# Prevalence and factors associated with hypertension among individuals undergoing premarital examination in Guangxi, China: a large cross-sectional study, 2022–2024

**DOI:** 10.3389/fpubh.2026.1948319

**Published:** 2026-10-06

**Authors:** BinLin Chen, SuHua Xia, YuHua Deng, Jing Huang, MingMing Zhang

**Affiliations:** 1 Department of Clinical Nutrition, Maternal and Child Health Hospital of Guangxi Zhuang Autonomous Region, Nanning, China; 2 Department of Women’s Health, Maternal and Child Health Hospital of Guangxi Zhuang Autonomous Region, Nanning, China; 3 Department of Obstetrics, Maternal and Child Health Hospital of Guangxi Zhuang Autonomous Region, Nanning, China; 4 Medical Examination Center, Maternal and Child Health Hospital of Guangxi Zhuang Autonomous Region, Nanning, China

**Keywords:** alanine aminotransferase, body mass index, China, educational status, Guangxi, hypertension, premarital examination

## Abstract

**Background:**

Hypertension constitutes a leading global public health challenge, with early detection in young adults critical for primordial and primary prevention. Premarital health examinations represent an underutilized platform for population-based cardiovascular screening. This study aimed to estimate the prevalence of hypertension and identify associated factors among individuals undergoing premarital examination in Guangxi, China.

**Methods:**

This large cross-sectional study was conducted at the Maternal and Child Health Hospital of Guangxi Zhuang Autonomous Region from 2022 to 2024, enrolling 11,283 consecutive premarital participants aged 20–50 years (5,641 males, 5,642 females). Sociodemographic, anthropometric, and metabolic indicators were collected using standardized questionnaires, physical measurements, and fasting biochemical tests. According to local premarital health policy, fasting blood glucose was measured only in female participants. Hypertension was defined as SBP ≥ 140 mmHg and/or DBP ≥ 90 mmHg, or self-reported physician-diagnosed hypertension. Univariate tests, multivariate logistic regression and restricted cubic spline (RCS) models were applied to identify correlates and dose–response relationships.

**Results:**

The overall prevalence of hypertension was 7.68%, with a significantly higher rate in men (10.28%) than in women (5.09%). In univariate analysis, age, body mass index (BMI), fasting blood glucose (FBG), alanine aminotransferase (ALT), sex, BMI classification, alcohol drinking, education level, abnormal FBG, and abnormal ALT were significantly associated with hypertension in the total population (all *p* < 0.05). Multivariate regression identified overweight (OR = 1.88, 95%CI: 1.27–2.77, *p* = 0.002), obesity (OR = 3.90, 95%CI: 2.29–6.64, *p* < 0.001), lower education level (OR = 1.64, 95%CI: 1.13–2.38, *p* = 0.009), abnormal ALT (OR = 1.69, 95%CI: 1.12–2.56, *p* = 0.013) and annual age increment (OR = 1.04, 95%CI: 1.01–1.08, *p* = 0.011) as factors associated with higher odds of hypertension, while underweight was associated with lower odds (OR = 0.42, 95%CI: 0.19–0.93, *p* = 0.033). In sex-stratified analyses, abnormal fasting blood glucose was independently associated with higher odds of hypertension in women (OR = 1.96, 95%CI: 1.21–3.16, *p* = 0.006). RCS analyses confirmed positive dose–response correlations between BMI, ALT, age and the odds of hypertension (all P for overall <0.05).

**Conclusion:**

Hypertension imposes a non-negligible health burden among premarital adults in Guangxi, with a markedly higher prevalence observed in males. Overweight, obesity, elevated liver enzyme levels, low educational attainment, advancing age, and abnormal fasting glucose (in women) are key correlates of hypertension in this population. Routine premarital health services should integrate comprehensive cardiovascular risk screening covering blood pressure measurement and BMI assessment, with targeted health education, to support long-term cardiovascular health among young reproductive-age populations.

## 1 Introduction

Hypertension remains the leading preventable risk factor for the global burden of cardiovascular diseases, including coronary heart disease, stroke, heart failure, and chronic kidney disease, and contributes substantially to premature mortality and disability worldwide (1). According to the 2024 Chinese Guidelines for the Prevention and Treatment of Hypertension, the overall prevalence of hypertension among Chinese adults aged 18 years and older reached 27.5% (2). In recent decades, the epidemiological transition and rapid urbanization in China have led to a marked increase in hypertension prevalence, with a particularly pronounced rising trend among young and middle-aged individuals: between 1991 and 2015, the prevalence increased by 25.1, 87.4, and 144.4% in the 60–79, 40–59, and 20–39 age groups, respectively (3). These alarming trends underscore the urgent need for early detection and intervention in younger populations.

Young adulthood represents a critical developmental stage for the establishment of long-term lifestyle habits and cardiovascular health trajectories. However, hypertension in young adults is usually asymptomatic, resulting in low awareness, low diagnosis rate, and delayed intervention, which eventually lead to subclinical target organ damage and increased long-term cardiovascular risk (4). Furthermore, elevated preconception blood pressure has been associated with prolonged time to pregnancy and increased risk of infertility (5), highlighting the particular importance of blood pressure control in the reproductive-aged population. Early detection during this life stage offers a crucial window for primordial and primary prevention. In this context, premarital health examinations offer a unique opportunity to assess blood pressure among young reproductive-aged individuals who might otherwise remain outside routine healthcare settings. In the present study, we used the blood pressure measurements obtained during the premarital visit as the basis for defining hypertension status. This approach aligns with the screening purpose of the premarital examination and allows us to estimate the prevalence of elevated blood pressure in this population, while acknowledging that confirmatory repeated measurements are beyond the scope of this cross-sectional investigation.

Guangxi Zhuang Autonomous Region is a multi-ethnic region in southern China with distinctive geographical conditions, dietary cultures and lifestyle patterns, which are known to shape the regional epidemiological characteristics of hypertension (6). A recent cross-sectional study covering the general population aged ≥18 years in Guangxi reported a hypertension prevalence of 39.7% (7), exceeding the national average and indicating a substantial cardiovascular disease burden in the local adult population. Despite this high background prevalence, there is a paucity of recent large-sample cross-sectional data regarding hypertension prevalence and associated factors specifically among local marriage-age relatively young people over the past 5 years, which constitutes a key innovation of the present study.

Against this backdrop, we conducted this large-sample cross-sectional study based on consecutive premarital examinees from 2022 to 2024 in Guangxi. This study aimed to estimate the prevalence of hypertension, identify independent factors associated with hypertension, and explore the dose–response relationships between key indicators and hypertension risk in this relatively young (20–50 years) reproductive-aged population. The findings are expected to provide high-quality real-world evidence for formulating regionally targeted hypertension prevention strategies and integrating comprehensive cardiovascular health management into routine premarital healthcare services.

## 2 Methods

### 2.1 Study design and study population

This was a large-sample cross-sectional study conducted at the Maternal and Child Health Hospital of Guangxi Zhuang Autonomous Region from January 1, 2022, to December 31, 2024. This hospital is a Grade III-A (tertiary) hospital located in Nanning, the capital of Guangxi, and provides premarital health examinations for couples of reproductive age from across the region; couples could freely choose any premarital examination institution in Guangxi. All individuals who underwent standard premarital health examinations were consecutively enrolled. Participants were eligible if they were aged 20–50 years and were Guangxi household-registered residents or had resided in Guangxi for at least 6 months; pregnant women were excluded. A total of 11,288 participants (5,644 couples) met the eligibility criteria. Of these, 5 participants with missing data on blood pressure, BMI, or ALT were excluded. Ultimately, 11,283 participants (5,641 males and 5,642 females) were included in the final analysis. The participant flow diagram is shown in Figure 1. Information on pre-existing cardiovascular disease, chronic kidney disease, or current antihypertensive medication use was not available in the routine premarital examination records; therefore, these conditions were neither exclusion criteria nor explicitly accounted for in the analysis, which is acknowledged as a limitation in the discussion.

**Figure 1:**
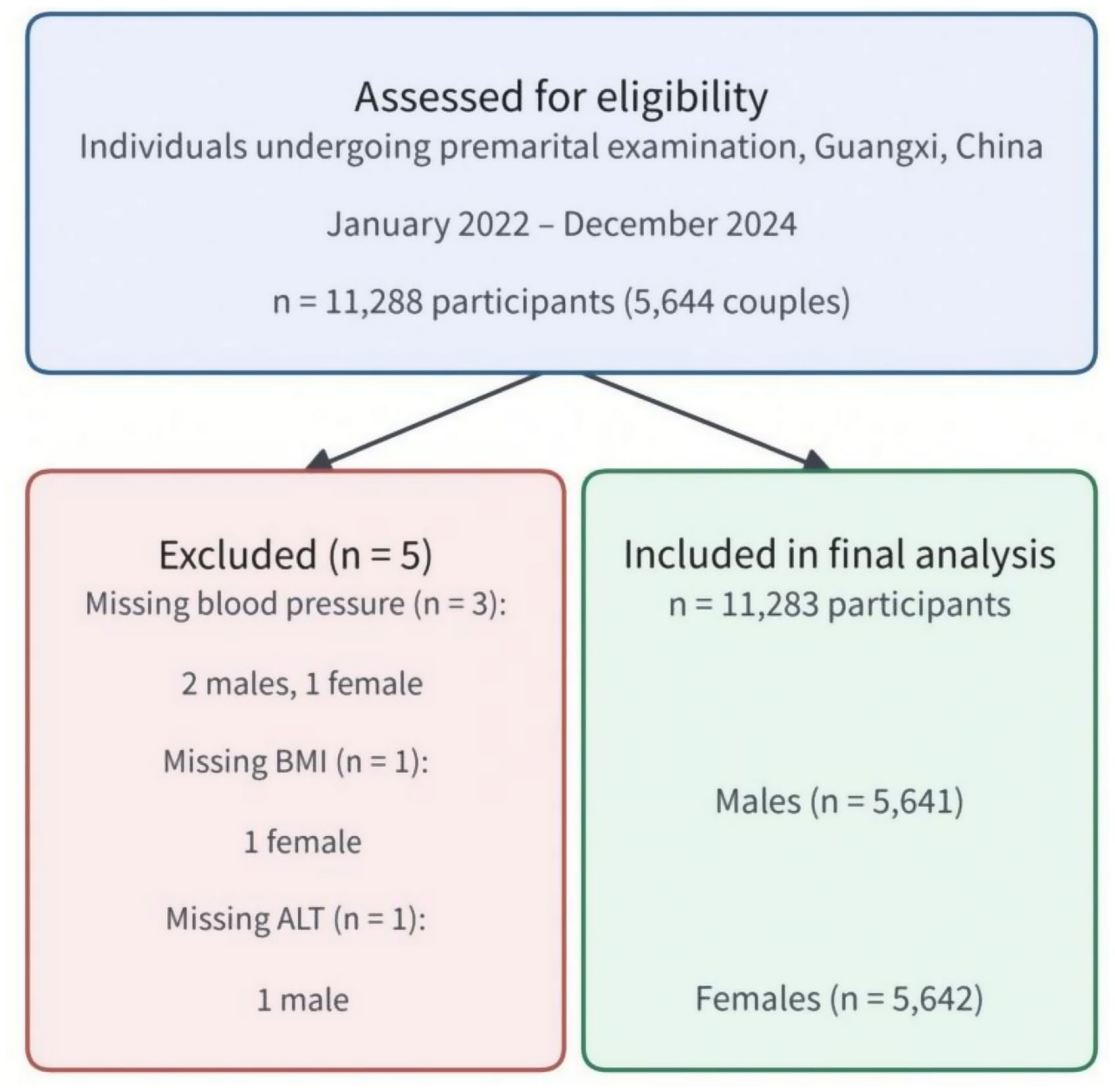
Flowchart of the sample inclusion process.

The study protocol was approved by the Ethics Committee of Maternity and Child Health Care of Guangxi Zhuang Autonomous Region (Approval No. [2026-5]7) and strictly adhered to the ethical principles outlined in the Declaration of Helsinki. Since all data were extracted from de-identified routine physical examination records without additional invasive examinations or collection of personal sensitive information, a waiver of written informed consent was approved by the ethics committee.

### 2.2 Data collection

Trained medical staff collected all data using a standardized questionnaire and physical examination form. The collected data included the following four parts:

Sociodemographic characteristics: age, sex, education level (college or above vs. high school or below), and alcohol drinking (yes vs. no). Alcohol drinking was defined as self-reported consumption of any alcoholic beverage at least once per week during the past year.

Anthropometric measurements: height, weight, and body mass index (BMI). Height and weight were measured by trained staff using a calibrated instrument (model BSM370, InBody Co., Ltd.), with height measured via an automatic touch bar to ensure accuracy. Height was recorded in centimeters (cm) to one decimal place, and weight was recorded in kilograms (kg) to one decimal place. BMI was calculated as weight in kilograms divided by height in meters squared (kg/m^2^). According to the Chinese criteria for adults (WS/T 428-2013), BMI was classified into four categories: underweight (<18.5 kg/m^2^), normal (18.5–23.9 kg/m^2^), overweight (24.0–27.9 kg/m^2^), and obesity (≥28.0 kg/m^2^).

Blood pressure measurement: Blood pressure was measured using a validated tunnel-type automated electronic blood pressure monitor (Up-Load Blood Pressure Monitor, model BPBIO320, InBody Co., Ltd.), which had been calibrated according to the manufacturer’s recommendations. The device employs an integrated compressor belt within a tunnel-type design, accommodating a range of upper-arm circumferences without requiring manual cuff size selection. All measurements were performed by trained medical staff who had received standardized training in blood pressure measurement procedures. Participants were instructed to avoid smoking, consuming caffeinated beverages or alcohol, and engaging in vigorous exercise for at least 30 min prior to the examination. Participants were seated in a quiet room with their back supported, feet flat on the floor, and legs uncrossed. After at least 5 min of rest, the participant’s right arm was inserted into the tunnel of the device at heart level, with the palm facing upward and the arm muscles relaxed. Two measurements were taken at an interval of 1 min, and the average value was used for analysis. If the difference between the two readings exceeded 10 mmHg for either systolic or diastolic pressure, a third measurement was performed, and the average of the latter two readings was recorded. All blood pressure measurements were performed in the morning (8:00–12:00), before fasting blood sampling. Hypertension was defined as SBP ≥ 140 mmHg and/or DBP ≥ 90 mmHg, or self-reported physician-diagnosed hypertension. Because blood pressure was assessed during a single premarital examination, the outcome should be interpreted as screening-detected hypertension/elevated blood pressure rather than confirmed clinical hypertension; participants with elevated measurements would ordinarily require repeat measurements on separate occasions to establish a clinical diagnosis.

Biochemical indicators: Fasting blood glucose (FBG) and alanine aminotransferase. (ALT) were tested using standard laboratory methods. FBG was tested only in female participants as per the local premarital examination protocol; accordingly, all FBG related analyses were restricted to women (*n* = 5,642), and denominators for FBG variables reflect the female sample. Abnormal FBG was defined as FBG ≥ 6.1 mmol/L according to the diagnostic criteria of impaired fasting glucose. Abnormal ALT was defined as ALT >40 U/L in females and ALT >50 U/L in males according to Chinese clinical diagnostic criteria.

### 2.3 Statistical analysis

All statistical analyses were performed using IBM SPSS Statistics 26.0 software and R 4.4.1. Continuous variables were tested for normality using the Shapiro–Wilk test. Variables with non-normal distribution were expressed as median (interquartile range, IQR: Q1, Q3) and compared between groups using Mann–Whitney U test; variables with normal distribution were expressed as mean ± standard deviation (SD) and compared using independent samples *t*-test. Categorical variables were presented as frequencies and percentages (*n*, %) and compared using *χ*^2^ test. Multivariable logistic regression was used to identify factors associated with hypertension. All binary covariates (sex, alcohol drinking, BMI classification, education level, and abnormal ALT) and age (continuous) were entered into the multivariable model as independent variables. To avoid data-driven selection, no stepwise procedure was applied, and all variables were retained in the final model irrespective of their univariate *p* values, in accordance with *a priori* clinical and epidemiological considerations. Results were reported as odds ratios (OR) with 95% confidence intervals (CI) and corresponding *p* values. Model adequacy was assessed using variance inflation factors (VIF) to evaluate multicollinearity, the Hosmer–Lemeshow goodness-of-fit test, and the area under the receiver operating characteristic curve (AUC) as a measure of discrimination. To assess effect modification by sex, multiplicative interaction terms (sex × education, sex × BMI, sex × abnormal ALT, and sex × age) were added separately to the main multivariable model and tested using the Wald test. To explore the dose–response relationships between continuous variables and the odds of hypertension, restricted cubic spline (RCS) regression with four knots at the 5th, 35th, 65th, and 95th percentiles was conducted to model the association of continuous BMI, ALT, and age with the odds of hypertension. The primary prespecified analyses were the estimation of hypertension prevalence and the multivariable logistic regression in the total population; the interaction tests and the RCS dose–response analyses were exploratory. Because multiple testing was performed, findings from the exploratory analyses should be interpreted with caution due to the increased risk of type I error. Two-sided *p* < 0.05 was considered statistically significant.

## 3 Results

### 3.1 Basic characteristics of the study population

A total of 11,283 participants were included in this study, with 5,641 men (50.00%) and 5,642 women (50.00%). The median age of the total population was 31.00 (29.00, 35.00) years, ranging from 22 to 50 years; the median age was 32.00 (30.00, 36.00) years in men (range 24–50 years) and 30.00 (28.00, 33.00) years in women (range 22–48 years). The overall prevalence of hypertension was 7.68% (867/11,283). The prevalence in men was 10.28% (580/5,641), which was significantly higher than the 5.09% (287/5,642) observed in women (*χ*^2^ = 107.32, *p* < 0.001).

### 3.2 Univariate analysis of factors associated with hypertension in the total, male, and female populations

Univariate analysis showed that, compared with normotensive participants, hypertensive participants were older, had higher BMI, higher ALT, and, in women, higher FBG levels (all *p* < 0.001). The proportions of overweight, obesity, lower education level, and abnormal ALT were significantly higher in the hypertensive group (all *p* < 0.05). Alcohol consumption was significantly more prevalent among hypertensive participants in the total population (*p* = 0.006), whereas the difference was not statistically significant within men (*p* = 0.200) or women (*p* = 0.684) separately. BMI classification was also significantly associated with hypertension (*p* < 0.001). Detailed results are presented in Table 1, which combines the total-population and sex-stratified univariate comparisons to allow direct visualization of the similarities and differences between men and women.

**Table 1:** Univariate analysis of factors associated with hypertension in the total, male, and female populations.

Variables	Total (*n* = 11,283)			Men (*n* = 5,641)			Women (*n* = 5,642)		
	Normotensive group (*n* = 10,416)	Hypertension group (*n* = 867)	*p*	Normotensive group (*n* = 5,061)	Hypertension group (*n* = 580)	*p*	Normotensive group (*n* = 5,355)	Hypertension group (*n* = 287)	*p*
Age, *M* (Q_1_, Q_3_)	31.00 (29.00, 34.00)	33.00 (30.00, 37.25)	**<0.001**	32.00 (29.00, 35.00)	33.50 (31.00, 38.00)	**0.002**	30.00 (28.00, 33.00)	31.00 (28.00, 36.00)	0.144
Height (cm), *M* (Q_1_, Q_3_)	165.00 (158.00, 170.00)	168.00 (160.00, 172.00)	**<0.001**	170.00 (168.00, 175.00)	170.00 (168.00, 175.00)	0.239	158.00 (155.00, 162.00)	158.00 (155.00, 162.00)	0.988
Weight (kg), *M* (Q_1_, Q_3_)	60.00 (50.00, 69.00)	70.00 (60.00, 80.00)	**<0.001**	68.00 (60.00, 75.00)	75.00 (66.00, 84.00)	**<0.001**	51.00 (47.00, 56.00)	56.00 (50.00, 65.00)	**<0.001**
BMI (kg/m^2^), *M* (Q_1_, Q_3_)	21.60 (19.60, 24.10)	24.80 (22.00, 27.70)	**<0.001**	23.20 (21.10, 25.40)	25.60 (23.40, 28.40)	**<0.001**	20.30 (18.80, 22.20)	22.30 (19.85, 25.65)	**<0.001**
FBG (mmol/L), *M* (Q_1_, Q_3_)	Only female testing			**–**	**–**		4.63 (4.24, 5.03)	4.83 (4.44, 5.29)	**<0.001**
ALT (U/L), *M* (Q_1_, Q_3_)	18.00 (12.00, 29.00)	28.00 (16.50, 47.50)	**<0.001**	25.00 (17.00, 39.00)	35.50 (22.00, 56.00)	**<0.001**	13.00 (10.00, 18.00)	16.00 (11.00, 25.50)	**<0.001**
Drinking alcohol, *n*(%)			**0.006**			0.200			0.684
No	7,596 (72.93)	595 (68.63)		2,852 (56.35)	343 (59.14)		4,744 (88.59)	252 (87.80)	
Yes	2,820 (27.07)	272 (31.37)		2,209 (43.65)	237 (40.86)		611 (11.41)	35 (12.20)	
BMI classification, *n*(%)			**<0.001**			**<0.001**			**<0.001**
Normal	6,366 (61.12)	337 (38.87)		2,784 (55.01)	176 (30.34)		3,582 (66.89)	161 (56.10)	
Underweight	1,316 (12.63)	38 (4.38)		225 (4.45)	11 (1.90)		1,091 (20.37)	27 (9.41)	
Overweight	2,178 (20.91)	289 (33.33)		1,625 (32.11)	231 (39.83)		553 (10.33)	58 (20.21)	
Obesity	556 (5.34)	203 (23.41)		427 (8.44)	162 (27.93)		129 (2.41)	41 (14.29)	
Education classification, *n*(%)			**<0.001**			0.727			**<0.001**
College or above	8,359 (80.25)	647 (74.63)		3,855 (76.17)	438 (75.52)		4,504 (84.11)	209 (72.82)	
High school or below	2057 (19.75)	220 (25.37)		1,206 (23.83)	142 (24.48)		851 (15.89)	78 (27.18)	
FBG, *n*(%)									**0.006**
Normal	Only female testing			**–**	**–**		5,115 (95.52)	264 (91.99)	
Abnormal				**–**	**–**		240 (4.48)	23 (8.01)	
ALT, *n*(%)			**<0.001**			**<0.001**			**<0.001**
Normal	9,304 (89.32)	645 (74.39)		4,211 (83.20)	392 (67.59)		5,093 (95.11)	253 (88.15)	
Abnormal	1,112 (10.68)	222 (25.61)		850 (16.80)	188 (32.41)		262 (4.89)	34 (11.85)	

Data are presented as median (Q_1_, Q_3_) for continuous variables or *n*/*N* (%) for categorical variables. For fasting blood glucose (FBG) variables, denominators reflect the female sample only (*N* = 5,642), as FBG was measured exclusively in women. *p*-values from Mann–Whitney *U* test or *χ*^2^ test. *Z*: Mann–Whitney test, *χ*^2^: Chi-square test. *M*: Median, Q_1_: 1st Quartile, Q_3_: 3rd Quartile. – indicates the value was not applicable; *p* values in bold indicate statistically significant differences (*p* < 0.05).

### 3.3 Multivariable logistic regression analysis in the total population and stratified by sex

For the multivariable logistic regression, all binary covariates (sex, alcohol drinking, BMI classification, education level, and abnormal ALT) and age as a continuous variable were entered into the model as independent variables, without stepwise selection. In the total population, the adjusted model showed that overweight (OR = 1.88, 95%CI: 1.27–2.77, *p* = 0.002), obesity (OR = 3.90, 95%CI: 2.29–6.64, *p* < 0.001), lower education level (OR = 1.64, 95%CI: 1.13–2.38, *p* = 0.009), abnormal ALT (OR = 1.69, 95%CI: 1.12–2.56, *p* = 0.013), and each 1-year increment in age (OR = 1.04, 95%CI: 1.01–1.08, *p* = 0.011) were associated with higher odds of hypertension. In contrast, underweight was associated with lower odds of hypertension (OR = 0.42, 95%CI: 0.19–0.93, *p* = 0.033). Sex and alcohol drinking were not independently associated with hypertension after adjustment (all *p* > 0.05). Detailed results are presented in Table 2.

**Table 2:** Multivariable logistic regression of factors associated with hypertension in the total population.

Variables	*β*	S.E.	*Z*	*p*	OR (95%CI)
Intercept	−4.30	0.59	−7.35	**<0.001**	0.01 (0.00–0.04)
Sex					
Male					1.00 (Reference)
Female	0.28	0.19	1.45	0.146	1.32 (0.91–1.92)
Drinking					
No					1.00 (Reference)
Yes	0.12	0.20	0.61	0.539	1.13 (0.76–1.67)
BMI classification					
Normal					1.00 (Reference)
Underweight	−0.86	0.40	−2.13	**0.033**	0.42 (0.19–0.93)
Overweight	0.63	0.20	3.16	**0.002**	1.88 (1.27–2.77)
Obesity	1.36	0.27	5.00	**<0.001**	3.90 (2.29–6.64)
Education classification					
College or above					1.00 (Reference)
High school or below	0.50	0.19	2.62	**0.009**	1.64 (1.13–2.38)
ALT					
Normal					1.00 (Reference)
Abnormal	0.52	0.21	2.49	**0.013**	1.69 (1.12–2.56)
Age	0.04	0.02	2.53	**0.011**	1.04 (1.01–1.08)

OR, Odds Ratio, CI, Confidence Interval. *p* values in bold indicate statistically significant differences (*p* < 0.05).

Because the prevalence of hypertension differed markedly between men and women, we further performed sex-stratified multivariable logistic regression analyses. The same core set of covariates (alcohol drinking, BMI classification, education level, abnormal ALT, and age) was entered separately for men and women; fasting blood glucose (FBG) was additionally included in the female model because FBG was measured only in women (Figure 2). In male participants (Figure 2A), overweight (OR = 2.05, 95%CI: 1.68–2.51, *p* < 0.001), obesity (OR = 3.97, 95%CI: 3.06–5.15, *p* < 0.001), lower education level (OR = 1.69, 95%CI: 1.39–2.04, *p* < 0.001), abnormal ALT (OR = 2.54, 95%CI: 2.09–3.08, *p* < 0.001), and each 1-year increment in age (OR = 1.06, 95%CI: 1.04–1.07, *p* < 0.001) were associated with higher odds of hypertension. Alcohol drinking (OR = 0.92, 95%CI: 0.76–1.10, *p* = 0.345) and underweight (OR = 0.59, 95%CI: 0.33–1.07, *p* = 0.085) were not significantly associated with hypertension. In female participants (Figure 2B), overweight (OR = 1.76, 95%CI: 1.22–2.54, *p* = 0.002), obesity (OR = 5.51, 95%CI: 3.16–9.61, *p* < 0.001), lower education level (OR = 1.57, 95%CI: 1.13–2.20, *p* = 0.008), abnormal FBG (OR = 1.96, 95%CI: 1.21–3.16, *p* = 0.006), and each 1-year increment in age (OR = 1.04, 95%CI: 1.01–1.07, *p* = 0.010) were associated with higher odds of hypertension, whereas underweight was associated with lower odds of hypertension (OR = 0.35, 95%CI: 0.20–0.61, *p* < 0.001). Alcohol drinking (OR = 1.41, 95%CI: 0.94–2.10, *p* = 0.094) and abnormal ALT (OR = 1.04, 95%CI: 0.64–1.68, *p* = 0.873) were not significantly associated with hypertension. These sex-stratified estimates are reported descriptively; their statistical comparability was assessed through interaction tests reported below.

**Figure 2:**
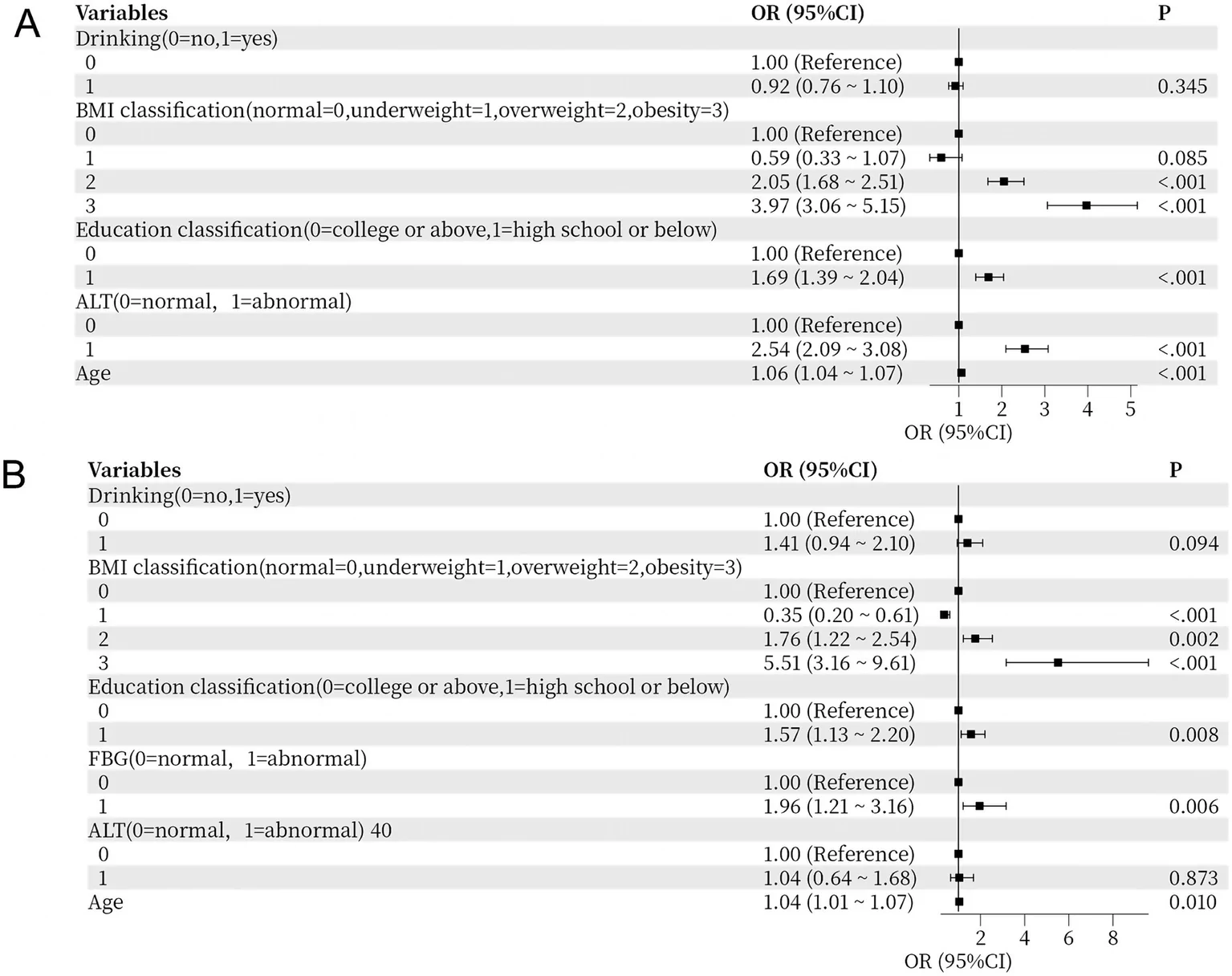
Forest plots of multivariable logistic regression for **(A)** male and **(B)** female participants.

To assess effect modification by sex, multiplicative interaction terms (sex × education, sex × BMI, sex × abnormal ALT, and sex × age) were added separately to the main multivariable model, which was adjusted for age, sex, BMI classification, abnormal ALT, alcohol drinking, and education level, as appropriate. None of the interaction terms was statistically significant. The sex × education interaction was not significant (interaction OR = 0.93, 95%CI: 0.44–1.95; *p* = 0.841 by both the Wald and likelihood ratio tests), indicating that the adjusted education–hypertension association was similar in direction and magnitude between men and women. Similarly, the sex × BMI (continuous BMI: OR = 1.03, 95%CI: 0.93–1.13, *p* = 0.582), sex × abnormal ALT (interaction OR = 0.61, 95%CI: 0.24–1.52; *p* = 0.289), and sex × age (interaction OR = 0.99, 95%CI: 0.93–1.06; *p* = 0.867) interactions were all non-significant. These results indicate that the associations of education, BMI, ALT, and age with hypertension were broadly similar between men and women, with no statistically significant effect modification by sex; apparent differences between the sex-stratified estimates should therefore be interpreted with caution.

Model adequacy diagnostics indicated satisfactory performance of the multivariable logistic regression models. For the total-population model, all variance inflation factors (VIF) were below 1.33 (maximum VIF = 1.33 for sex), indicating no meaningful multicollinearity; the Hosmer–Lemeshow test was non-significant (*χ*^2^ = 5.96, df = 8, *p* = 0.652), suggesting adequate goodness of fit; the area under the receiver operating characteristic curve (AUC) was 0.69, indicating acceptable discrimination. The sex-stratified models showed similarly adequate diagnostics (all VIF < 1.15; all Hosmer–Lemeshow *p* > 0.57; AUC 0.68–0.69).

### 3.4 Dose–response relationships between BMI, ALT, age and the odds of hypertension

Restricted cubic spline (RCS) analysis with four knots (5th, 35th, 65th, and 95th percentiles) was conducted to explore the dose–response relationships between continuous variables (BMI, ALT, age) and odds of hypertension. The results demonstrated positive associations for all three variables (all *P* for overall <0.001), with the odds of hypertension rising with increasing levels of BMI, ALT, and age (Figure 3). The BMI curve showed a significant nonlinear pattern (*P* for nonlinear <0.001). Similarly, the ALT curve also exhibited a significant nonlinear relationship (*P* for nonlinear <0.001). In contrast, the age curve demonstrated an approximately linear association (*P* for nonlinear = 0.354), indicating that the odds of hypertension increased steadily with each year of age, with a more pronounced upward trend particularly evident after the reference age of 31 years.

**Figure 3:**
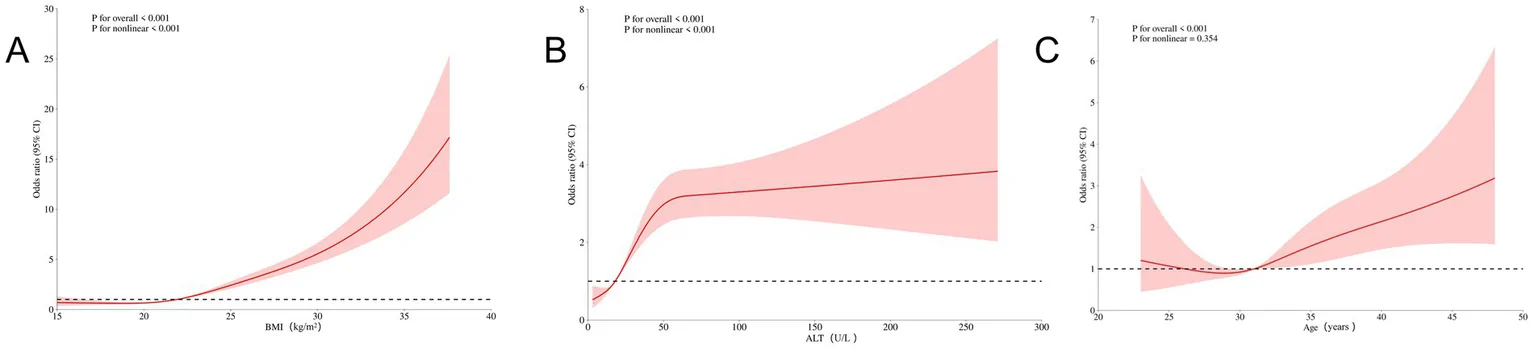
Restricted cubic spline (RCS) curves of the dose–response relationships between BMI, ALT, age and hypertension risk in the total premarital examination population. The RCS analysis was conducted with four knots at the 5th, 35th, 65th, and 95th percentiles of the respective variable distributions. The *X*-axis represents the continuous variable, and the *Y*-axis represents the odds ratio (OR) of hypertension risk relative to the reference value (dashed vertical line) of each variable. The shaded areas represent the 95% confidence intervals (95%CI) for the OR estimates. **(A)** BMI (knots: 17.6, 20.5, 23.3, 28.7 kg/m^2^; reference = 21.9 kg/m^2^); **(B)** ALT (knots: 8, 14, 24, 69 U/L; reference = 18 U/L); **(C)** age (knots: 26, 30, 33, 41 years; reference = 31 years).

## 4 Discussion

This large-scale cross-sectional study of 11,283 relatively young adults undergoing premarital health examinations in Guangxi, China, revealed a hypertension prevalence of 7.68%, with a notably higher prevalence in men (10.28%) than in women (5.09%). Our findings indicate that elevated BMI and abnormal ALT are independently associated with higher odds of hypertension in this population. In addition, lower education level was associated with higher odds of hypertension in both sexes.

The overall hypertension prevalence of 7.68% in this relatively young population (median age 31 years) is noteworthy and underscores the importance of early screening. This prevalence is lower than the 12.8% reported among Chinese adults aged 18–44 years in 2019 (8), as well as the 14.3% observed among US adults aged 18–39 years (9). Although the prevalence in our study population is comparatively lower than these estimates, the high background prevalence of 39.7% among Guangxi adults aged ≥18 years highlights that young reproductive-aged individuals—who are generally healthy and actively seeking premarital health certification—represent a critical window for primordial and primary prevention of hypertension (7). The higher prevalence in men is consistent with global and national trends (10, 11), which may be attributed to a combination of biological factors (e.g., protective effects of estrogen in premenopausal women) and behavioral factors (e.g., higher rates of smoking, alcohol consumption, and occupational stress among men) (12).

Our study found that overweight and obesity were associated with 1.88-fold and 3.90-fold higher odds of hypertension, respectively, in the total population. The RCS curves showed a positive dose–response relationship between BMI and the odds of hypertension. The strong association between BMI and the odds of hypertension, as demonstrated by both logistic regression and RCS analysis, reinforces the important role of adiposity in blood pressure regulation. Our findings are consistent with extensive literature demonstrating that excess body weight increases sympathetic nervous system activity, activates the renin-angiotensin-aldosterone system, promotes insulin resistance, and induces chronic low-grade inflammation, all of which contribute to elevated blood pressure (13, 14). The association between underweight and lower odds of hypertension (OR = 0.42) further supports the inverse relationship between lower BMI and the odds of hypertension.

Elevated ALT, a marker of hepatocellular injury, was independently associated with higher odds of hypertension, consistent with prior evidence linking liver dysfunction to cardiovascular disease (15–17). RCS curves verified a progressive positive correlation between ALT level and the odds of hypertension. Non-alcoholic fatty liver disease (NAFLD) has been associated with hypertension in prior literature, potentially through shared mechanisms such as insulin resistance, systemic inflammation, and endothelial dysfunction (18, 19). Animal experiments have demonstrated bidirectional interactions between liver injury and hypertension: hypertensive status aggravates hepatic steatosis and fibrosis, while impaired liver function disrupts vascular homeostasis to raise blood pressure (20). Population-based cohort studies from Iran and other regions also supported that elevated liver enzymes independently predict incident hypertension and serve as auxiliary indicators for early hypertension screening (17, 21). In the sex-stratified regression, abnormal ALT was associated with higher odds of hypertension in the total population (OR = 1.69) and in men (OR = 2.54), but the association did not reach statistical significance in women (OR = 1.04, 95%CI: 0.64–1.68, *p* = 0.873), possibly reflecting the lower prevalence of both abnormal ALT and hypertension among women in this reproductive-aged sample. Nevertheless, ALT elevations identified during premarital examinations warrant additional metabolic and cardiovascular risk evaluation, particularly among those presenting with elevated blood pressure and/or overweight/obesity.

Age was identified as a factor associated with higher odds of hypertension in the total population and in both sexes. The RCS analysis supported these findings, showing an approximately linear positive association between age and the odds of hypertension in the total population. Advanced age has been repeatedly validated as a reliable marker associated with hypertension in domestic and international epidemiological studies (22, 23). Notably, in the univariate analysis, age did not differ significantly between hypertensive and normotensive women (*p* = 0.144), whereas in the multivariable model adjusted for BMI, education, ALT, drinking, and FBG, age emerged as a significant factor associated with higher odds of hypertension in women (OR = 1.04, 95%CI: 1.01–1.07, *p* = 0.010). This change from non-significant to significant after adjustment suggests that the crude age effect in women was masked by confounding from other covariates; once these factors were accounted for, the independent age association became detectable. The relatively narrow age range and younger median age (30 years) of female participants may have limited the statistical power in the univariate analysis, while hormonal factors in premenopausal women may be associated with a slower age-related increase in blood pressure (24, 25).

In the multivariable analysis, lower education level was associated with higher odds of hypertension after adjustment for BMI, sex, age, and other covariates in the overall population and in both sexes (men: OR = 1.69, 95%CI: 1.39–2.04, *p* < 0.001; women: OR = 1.57, 95%CI: 1.13–2.20, *p* = 0.008). This finding is consistent with studies demonstrating an inverse association between educational attainment and hypertension (26, 27). Using Demographic and Health Surveys from 21 low- and middle-income countries (2013–2023), Andriani et al. found that the burden of hypertension among women of reproductive age was driven by advanced age, lower education, high BMI, and rural residence (26). Similarly, a nationwide cross-sectional study in China reported that lower socioeconomic status—derived from educational attainment, living conditions, and marital status—was associated with a higher prevalence of hypertension (27). These observations highlight the importance of considering educational attainment and broader socioeconomic factors when designing targeted hypertension prevention strategies for young adults undergoing premarital examination.

Abnormal fasting blood glucose (FBG) was independently associated with higher odds of hypertension in women (OR = 1.96, 95%CI: 1.21–3.16, *p* = 0.006), even after adjustment for BMI, age, education, ALT, and alcohol consumption. This finding is consistent with accumulating evidence linking dysglycemia to elevated blood pressure (28, 29). A recent analysis demonstrated that diabetes diagnosed before age 40 years was associated with higher subsequent cardiovascular disease risk beyond established risk factors, with risk increasing as cumulative glucose exposure rose (30). Two meta-analyses demonstrated that diabetes approximately doubles vascular disease risk independently of conventional factors, and that fasting glucose is modestly associated with vascular and coronary heart disease risk even in non-diabetic individuals (31, 32). The observed association between abnormal FBG and higher odds of hypertension in our female premarital examination sample suggests that glucose assessment may provide additional information for cardiovascular risk evaluation among young women, although prospective studies are needed to confirm its clinical utility in this setting.

Although alcohol consumption was correlated with hypertension in univariate analysis, this association lost statistical significance after multivariate adjustment. The crude correlation may be confounded by overlapping metabolic and demographic covariates such as BMI and sex, and our binary drinking definition could not capture detailed drinking patterns, which may partly explain the attenuated independent effect (33).

This study has several methodological strengths, including a large consecutive sample, sex-stratified analysis, RCS dose–response modelling, and interaction testing. Several limitations should nevertheless be acknowledged. First, the cross-sectional design cannot establish causal relationships between measured indicators and hypertension, and reverse causality or bidirectional associations cannot be excluded because exposure and outcome were assessed within the same examination period. Second, the study was conducted at a single maternal and child hospital, and individuals attending this particular premarital examination centre may differ systematically from those using other examination centres or those not undergoing premarital examinations; therefore, the prevalence estimate may not be directly generalizable to all young adults in Guangxi. Third, we lacked data on several important confounders, including smoking, physical activity, dietary sodium intake, sleep, and family history of hypertension. Their absence may have led to residual confounding. Fourth, hypertension was defined based on blood pressure measurements from a single visit (averaged from two readings), without confirmatory repeated measurements on separate occasions; the outcome should therefore be interpreted as screening-detected elevated blood pressure rather than confirmed clinical hypertension, and the observed prevalence may overestimate true clinical hypertension. Fifth, information on current antihypertensive medication use and pre-existing cardiovascular or renal disease was not available; because antihypertensive treatment can directly affect measured blood pressure and hypertension classification, this represents an additional source of potential misclassification. Sixth, FBG was measured only in female participants, limiting assessment of glucose-related associations in men. Seventh, participants were recruited as couples, and observations within couples may not be fully independent; however, no couple identifier was available in the de-identified dataset, which precluded cluster-robust standard errors, generalized estimating equations, or mixed-effects modelling to account for the paired structure. Finally, because multiple analyses were performed, the increased risk of type I error from multiple comparisons should be considered when interpreting the findings.

Our findings have important implications for public health practice. Premarital health examinations represent a critical and underutilized opportunity for early cardiovascular risk screening in young adults. The integration of blood pressure measurement and BMI assessment into routine premarital care could effectively identify high-risk individuals and facilitate early intervention. Additionally, ALT elevations and abnormal FBG identified during routine testing may serve as useful markers for further metabolic and cardiovascular risk stratification.

## 5 Conclusion

In this large cross-sectional study of premarital adults in Guangxi, China, hypertension prevalence was 7.68%, substantially higher in men (10.28%) than women (5.09%). Elevated BMI and ALT showed dose-dependent associations with higher odds of hypertension; lower education was independently associated in both sexes, and abnormal fasting blood glucose in women. These findings underscore premarital examinations as a critical opportunity for early cardiovascular risk screening—including blood pressure, BMI, and glucose assessment—with targeted health education to benefit the next generation.

## Data Availability

The original contributions presented in the study are included in the article/supplementary material, further inquiries can be directed to the corresponding author/s.
